# Impact of the omicron phase on a highly advanced medical facility in Japan

**DOI:** 10.3389/fped.2023.1201825

**Published:** 2023-07-11

**Authors:** Hirokazu Yamagishi, Daisuke Tamura, Jun Aoyagi, Shun Suzuki, Yoshitaka Mizobe, Keizo Wakae, Takanori Yamagata, Toshihiro Tajima, Hitoshi Osaka

**Affiliations:** Department of Pediatrics, Jichi Medical University, Shimotsuke-shi, Japan

**Keywords:** coronavirus disease 2019, pediatric, febrile seizure, severe illness rate, the hospitalization rate

## Abstract

**Background:**

Eight waves of the coronavirus disease 2019 (COVID-19) epidemic have been observed in Japan. This retrospective study was conducted to clarify the clinical characteristics of pediatric COVID-19 patients.

**Methods:**

We studied 121 patients admitted to the Jichi Children's Medical Center Tochigi between April 2020 and March 2023. Incidence of pediatric COVID-19 in Tochigi Prefecture was used to examine hospitalization and severe illness rates.

**Results:**

The mean age of the patients was 3 years and 8 months. One hundred and eleven patients (91.7%) were hospitalized after January 2022 (after the 6th wave), when the Omicron strain became endemic in Japan. Convulsions occurred in 30 patients (24.8%), all of whom were admitted after the 6th wave. Twenty-three of the 30 patients had no underlying disease. Eleven patients (9.1%) were diagnosed with acute encephalopathy. One patient died due to hemorrhagic shock and encephalopathy syndrome and two had sequelae after the 6th wave. The patient who died due to encephalopathy had hypercytokinemia. In the Tochigi Prefecture, the number of pediatric COVID-19 patients increased after the 6th wave, but the hospitalization rate declined. The rate of severe illness did not change before the end of 5th and after the 6th wave.

**Conclusion:**

Although the rate of severe illness in patients with pediatric COVID-19 did not increase after the 6th wave, some patients had complicated critical illnesses. Systemic inflammatory reaction was considered to have been associated with the severe encephalopathy.

## 1. Introduction

The novel coronavirus disease 2019 (COVID-19) remains out of control and is spreading worldwide (1). Japan had seven major outbreaks before October 2022, and the eighth major outbreak began in November 2022 (2). Several strains of the severe acute respiratory syndrome coronavirus-2 (SARS-CoV-2) have been recorded since its first report in late 2019 (1). In Japan, the Wuhan strain was prevalent during the first wave from March to June 2020 and the second wave from July to October 2020. Alpha, Beta, and Gamma strains were identified in the third wave, from October 2020 to February 2021. In the fourth wave, from March to May 2021, alpha strains accounted for the majority. The Delta strains were first identified in March 2021 and then became the major endemic strains in the fifth wave from June to December 2021. Omicron strains have been endemic since the sixth wave, which began in January 2022 (3). The number of infected persons by age group was 5%–15% for those under 20 years of age before the 4th wave. However, it has increased to approximately 20% and 30% in the 5th and 6th waves, respectively (2).

In a report discussing the severity of pediatric COVID-19 hospitalizations up to the first half of the sixth wave in Japan, Shoji et al. summarized that most hospitalized pediatric patients with COVID-19 were mild cases (4, 5). However, this report did not examine detailed blood data or their courses of treatment after hospitalization. In addition, because only hospitalized patients were enrolled in these reports, the severe illness rate presented did not represent the overall severity rate of SARS-CoV-2-positive cases.

Tochigi Prefecture has a total population of 1.9 million, with 230,000 comprising those aged <15 years. The Jichi Children's Medical Center Tochigi (JCMCT) is the only highly advanced pediatric medical facility in the Tochigi Prefecture with Pediatric Intensive Care Unit (PICU), equipped with extracorporeal membrane oxygenation (ECMO) for children. The JCMCT accepts patients in Tochigi Prefecture who require advanced medical care for congenital heart disease, hematologic malignancies, immunodeficiency, neurological diseases, and other pediatric patients with severe COVID-19 who are difficult to treat at other facilities. In addition, the JCMCT accepts mild or asymptomatic patients if they are requested by the local government and/or referred by other hospitals. No report has summarized the details of the epidemic situation of pediatric patients with COVID-19 in the region and clarified the acceptance and subsequent clinical course of pediatric COVID-19 cases at the core pediatric highly advanced medical facility in the region under such circumstances.

In this study, we conducted a retrospective analysis of pediatric patients with COVID-19 admitted to the JCMCT, a highly advanced pediatric care facility, to clarify their clinical characteristics. Additionally, we aimed to clarify the hospitalization and severity rates for pediatric COVID-19 in Tochigi Prefecture, where several large epidemics with different mutant strains have circulated.

## 2. Patients and methods

### 2.1. Study design and data participants

This was a retrospective study based on the clinical records of hospitalized patients. The study included patients admitted to JCMCT between March 1, 2020, and March 31, 2023, who tested positive for SARS-CoV-2 at the time of admission.

### 2.2. Definition of severity

The National Institute of Health (NIH) COVID-19 treatment guidelines have been used to assess severity (6). NIH criteria are based primarily on the intensity of respiratory impairment caused by COVID-19. However, in this study, because there were cases of acute encephalopathy and myocarditis requiring intensive care even in the absence of severe pneumonia, the following definitions were used along with the NIH criteria, referring to the guidelines for acute encephalopathy (7) and those proposed for COVID-19-related myocarditis (8). The criteria from the Council of State and Territorial Epidemiologists and Center for Disease Control and Prevention were used to define multisystem inflammatory syndrome in children (MIS-C) (9) (Table 1).

**Table 1:** Definition of severity.

Asymptomatic (6)	Only positive virological (nucleic acid amplification or antigen test) SARS-CoV-2, without symptoms consistent with COVID-19.
Mild illness (6)	With symptoms such as fever >37.5°C, cough without dyspnea caused by COVID-19, and no decrease in saturation of oxygen (SpO2) under 98%.
Moderate illness (6–8)	With findings of lower respiratory tract inflammation and SpO2 >94%, with seizure clusters or status epilepticus but showing improvement in impaired consciousness within 24 h, and with chest pain associated with myocarditis.
Severe illness (6–8)	Lower respiratory tract inflammation and SpO2 <94%, ratio of arterial partial pressure of oxygen to fraction of inspired oxygen (PaO2/FiO2) <300 mmHg, and Glasgow coma scale (GCS) <12 with prolonged disturbance of consciousness for more than 24 h, or with circulatory instability due to arrhythmia.
Critical illness (6)	With respiratory failure or impaired consciousness, requiring intubated ventilator management, sepsis, or multiorgan failure.
MIS-C (9)	Age <21 years old, Fever ≥38.0°C, Clinical severity requiring hospitalization or resulting in death, C-reactive protein ≥3.0 mg/dl, New onset manifestations in at ≥2 of the following category a–e; a. Cardiac involvement such as left ventricular ejection fraction <55%, troponin elevated, and so on; b. Mucocutaneous involvement such as rash, redness of the eyes, and so on; c. Shock; d. Gastrointestinal involvement; e. Hematologic involvement. AND; Detection of SARS-CoV-2 RNA, or specific antigen in a clinical specimen up to 60 days prior to hospitalization. OR; Detection of SARS-CoV-2 specific antibodies in serum, plasma or whole blood associated with current illness resulting in or during hospitalization.

MIS-C, multisystem inflammatory syndrome in children.

Asymptomatic and mild illnesses were classified as the mild group. Because similar to severe illnesses, moderate illnesses required management to prevent exacerbation of symptoms and firm follow-up, moderate, severe, and critical illnesses were classified as the severe group.

In the case of patients with chronic lung disease or cyanotic heart disease, if their SpO2 falls below 94% on room air under normal circumstances, the inability to maintain their usual SpO2 levels due to COVID-19 is indicative of a severe illness.

Since the population of young patients has been increasing and Omicron strains have become the predominant strain since the 6th wave, patients in this study were divided into two groups: the pre-6th wave group, hospitalized before December 2021, and the post-6th wave group, hospitalized after January 2022.

### 2.3. Data collection

Data on sex, age, underlying disease, date of admission, symptoms at admission, and blood test data at admission [white blood cells (WBC), neutrophils (Neutr), lymphocytes (Lymph), platelet cells (PLT), D-dimer, serum C-reactive protein (CRP), creatinine (Cr), aspartate aminotransferase (AST), alanine aminotransferase (ALT), lactate dehydrogenase (LDH), creatine kinase (CK), procalcitonin (PCT)], records of diagnosis at admission, treatment details, length of hospital stay, and outcomes were collected.

Information regarding the number of pediatric patients with COVID-19 and overall hospitalized patients in Tochigi Prefecture during the study period was collected from the Medical Countermeasures Team, Headquarters for Novel coronavirus infectious Disease Control, Government of Tochigi Prefecture.

### 2.4. Mutation analysis

Mutation analysis of SARS-CoV-2 was performed for patients who provided informed consent. SARS-CoV-2 Direct Detection RT-qPCR Core Kit and Primer/Probe N501Y (SARS-CoV-2), Primer/Probe L452R (SARS-CoV-2), and Primer/Probe E484A (Shimadzu, Kyoto, Japan) were used for reverse transcription reactions (5 min at 52°C, 10 s at 95°C) and 40 cycles PCR (5 s at 95°C, 30 s at 60°C) to determine the presence of *N501Y* (e.g., Alpha or Beta strain), *L452R* (e.g., Delta strain), or *E484A* (e.g., Omicron strain) mutations in the *S* gene encoding the viral spike protein.

### 2.5. Statistical analysis

Comparison of the admission rates to the JCMCT between the pre-6th wave and post-6th wave groups against the number of pediatric COVID-19 admissions in Tochigi Prefecture; comparison of the hospitalization rates of pediatric patients with COVID-19 in Tochigi Prefecture, the pre-6th wave group, and the post-6th wave group; comparison of the admission rates to the JCMCT for pediatric patients with COVID-19 in Tochigi Prefecture, the pre-6th wave group, and the post-6th wave group; comparison of the proportion of severe cases by sex and presence or absence of underlying disease; and comparison of the background factors, case symptoms, diagnosis, and treatment for each case between the pre-6th wave and post-6th wave groups, were performed using the chi-squared test.

Fisher's exact test was used if the expected value was <5. Additionally, comparison of the proportion of severe cases between the pre-6th wave and post-6th wave groups, and comparison of the incidence rates of severity in the pre-6th and post-6th waves among pediatric patients with COVID-19 in Tochigi Prefecture were performed using Fisher's exact test. Meanwhile, comparisons of the blood data between the mild and severe groups, and between patients with and without neurological symptoms were performed using the Mann–Whitney *U*-test.

All statistical analyses were performed using R statistical software (https://cran.r-project.org) version 4.1.2.

### 2.6. Ethics statement

This study was approved by the Ethics Committee of the Jichi Medical University Hospital (22-133; approval date January 23, 2023). Written informed consent was obtained from all participants or their parents.

## 3. Results

### 3.1. Profiles of patients

One hundred and twenty-one patients tested positive for SARS-CoV-2 on admission during the study period, with 61 of them (50.4%) being male patients. The mean age was 3 years and 8 months (range: 0 months to 32 years), and 106 patients were under 15 years old. Fifty-eight patients (47.9%) had underlying diseases. Thirty-six patients (29.8%) had underlying neurological disease such as cerebral palsy, and epilepsy, 15 (12.4%) patients had cardiovascular disease such as Fallot's tetralogy and single ventricle, and 6 (4.9%) patients had respiratory disease, including chronic lung disease and airway stenosis. Eleven patients (9.1%) had multiple organ disorders associated with chromosomal or genetic abnormalities (Table 2). Five patients had been using home oxygen therapy before the onset of COVID-19 due to chronic lung disease or cyanotic heart disease, and a patient with Apert syndrome was using a high-flow nasal cannula due to concomitant upper airway stenosis. Two patients with cerebral palsy and one patient with trisomy 18 underwent tracheostomy and home ventilation for chronic respiratory failure. One patient was using tacrolimus after liver transplantation for biliary atresia, one patient with refractory nephrotic syndrome was treated with steroids and mizoribine, one patient with B-cell leukemia was treated with oral mercaptopurine hydrate for 3 weeks before the onset of COVID-19, and one patient with spinal medulloblastoma was treated with intravenous bevacizumab, irinotecan hydrochloride, and oral temozolomide.

**Table 2:** Patient demographics.

		*N*	(%)
Sex	Male	60	(49.6)
Age	Under 1 year old	26	(21.5)
	1–4 years old	40	(33.1)
	5–11 years old	40	(33.1)
	12–15 years old	7	(5.8)
	Over 16 years old	8	(6.6)
Underlying disease		58	(47.9)
	Neurological disease	36	(29.8)
	Cardiovascular disease	15	(12.4)
	Respiratory disease	6	(5.0)
	Gastrointestinal disease	5	(4.1)
	Renal disease	3	(2.5)
	Metabolic disease	3	(2.5)
	Hematological disease	3	(2.5)

### 3.2. Domestic epidemic wave

**Figure 1:**
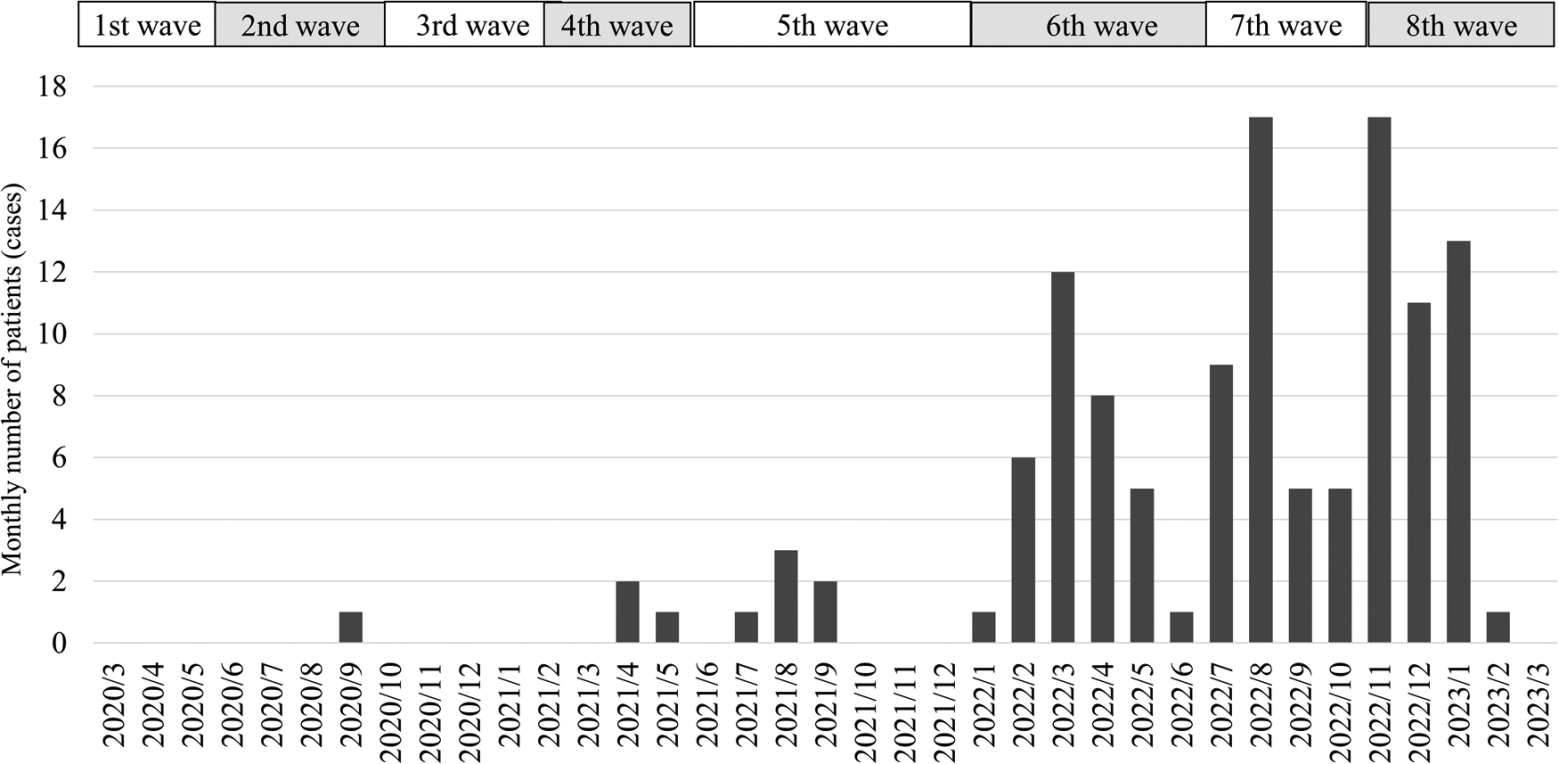
The monthly number of hospitalized patients at Jichi Children's medical center tochigi. The 1st wave lasted from March to June 2020. The 2nd wave lasted from June to October 2020. The 3rd wave lasted from October 2020 to February 2021. The 4th wave lasted from March to May 2021. The 5th wave lasted from June to December 2021. The 6th wave lasted from January to June 2022. The 7th wave lasted from July to October 2022. The 8th wave has lasted from November 2022. The number of COVID-19 patients admitted to the Jichi Children's Medical Center Tochigi from the 1st to the 5th wave was 10. Thirty-three patients were hospitalized in the 6th wave and 36 in the 7th wave. In the 8th wave, 42 patients were hospitalized until March 2023.

The JCMCT had ten hospitalized patients with COVID-19 before the end of the 5th wave. In the sixth wave, the number of hospitalizations increased sharply to 33 patients; in the seventh wave, 36 patients; and in the eighth wave, 42 patients were hospitalized until March 2023 (Figure 1).

Before November 2022, for children under 15 years of age, we compared the number of admissions to our hospital (*n* = 91) with that in Tochigi Prefecture (no data on the number of patients aged <15 years admitted in Tochigi Prefecture after December 2022). Before the end of the 5th wave, there were 236 admissions of patients with COVID-19 in Tochigi Prefecture, of whom nine were admitted to the JCMCT. After the 6th wave (January–November 2022), the number of admissions to Tochigi Prefecture was 464, and the number of admissions to the JCMCT increased to 82. After the 6th wave (January–November 2022), the number of patients requiring hospitalization at the JCMCT was significantly higher (pre-6th wave vs. post-6th wave: 3.8% vs. 17.7%, *p* < 0.001).

For children aged ≤15 years, the total number of patients with COVID-19 in Tochigi Prefecture was 1,588 before the end of the 5th wave. After the 6th wave (January to September 2022, data were not available after October 2022 due to the completion of the total number of cases), the number was 51,081. The hospitalization rate for patients in Tochigi Prefecture dropped significantly after the 6th wave (pre-6th wave vs. post-6th wave: 1.5% vs. 0.9%, *p* < 0.001). The rate of hospitalization to the JCMCT for patients in Tochigi Prefecture also significantly decreased after the 6th wave (pre-6th wave vs. post-6th wave: 0.14% vs. 0.1%, *p* < 0.001).

### 3.3. Symptoms, diagnosis, and treatment upon admission

On admission, 108 (89.3%) patients had a fever >37.5°C, 42 (34.7%) had coughs, and 30 (24.8%) had convulsions. Acute upper respiratory tract infection was diagnosed in 71 (58.7%) patients, bronchitis in 12 (9.9%), and pneumonia in 5 (4.1%). Eleven patients (9.1%) had acute encephalopathy and were administered steroid pulse therapy, and one patient (0.8%) had myocarditis. Furthermore, intubation and ventilator management was performed in two (1.7%) of the patients with acute encephalopathy. The patient with HSES met the definition of MIS-C (Table 3).

**Table 3:** Symptoms, diagnosis, and treatment of the cases.

		*n*	(%)
Symptom	Fever >37.5°C	108	(89.3)
	Cough	42	(34.7)
	Convulsion	30	(24.8)
	Runny nose	17	(14.0)
	Sputum increase	15	(12.4)
	Impaired consciousness	15	(12.4)
	Diarrhea	10	(8.3)
	Vomit	10	(8.3)
	Sore throat	7	(5.8)
	Headache	5	(4.1)
	Chest pain	2	(1.7)
	Muscle pain	1	(0.8)
	Apnea	1	(0.8)
	Dysgeusia	1	(0.8)
	Without symptoms	4	(3.3)
Diagnosis	Upper respiratory infection	71	(58.7)
	Complex febrile seizure	15	(12.4)
	Bronchitis	12	(9.9)
	Acute encephalopathy	11	(9.1)
	Gastroenteritis	6	(5.0)
	Pneumonia	5	(4.1)
	Febrile delirium	4	(3.3)
	Simple febrile seizure	2	(1.7)
	Myocarditis	1	(0.8)
	Kawasaki disease	1	(0.8)
	Myositis	1	(0.8)
	Diabetic ketoacidosis	1	(0.8)
Treatment	Transfusion	89	(73.6)
	Antifebrile drug	60	(49.6)
	Expectorant drug	23	(19.0)
	Oxygenation	20	(16.5)
	Remdesivir	15	(12.4)
	Methylprednisolone pulse	11	(9.1)
	Intravenous immunoglobulins	3	(2.5)
	Intubation and respirator management	2	(1.7)
	High-flow nasal cannula	2	(1.7)
	Dexamethasone	1	(0.8)
	Percutaneous pacing	1	(0.8)
	Without treatments	18	(14.9)

The mean age of the 30 patients with convulsions was 4 years and 7 months, ranging from 11 months to 10 years and 10 months; 12 (40.0%) of the 30 patients were aged ≥5 years. Seven (23.3%) of the 30 patients had underlying neurological disease: four with epilepsy, two with cerebral abnormality such as brain tumor and ventriculomegaly, and one with autism spectrum disorder. The other 23 (76.7%) patients had no underlying disease. Sixteen patients (53.3%) had status epilepticus, and ten patients (33.3%) had seizure clusters. Of the patients aged ≥5 years, 12 had seizures, 7 (58.3%) had previous febrile convulsions or underlying epilepsy, whereas 5 (41.7%) had seizures for the first time.

All the 11 patients with acute encephalopathy had no underlying disease.

Five patients who had been on home oxygen therapy before admission for COVID-19 did not require an increase in oxygen dosage. Three patients on home ventilator did not require any changes in ventilator conditions before or after treatment.

One hundred and six patients (89.3%) underwent blood tests upon admission. The level of WBC was 7,442 ± 3,775/µl, neutrophils: 4,732 ± 3,287/µl, lymphocytes: 1,846 ± 1,630/µl, PLT: 231,000 ± 114,000/µl, D-dimer; 19.3 ± 83.9 µg/ml, CRP: 1.03 ± 1.9 mg/dl, Cr: 0.37 ± 0.21, 0.39 ± 0.23 mg/dl, AST: 59 ± 113 U/L, ALT: 34 ± 53 U/L, LDH: 351 ± 189 U/L, CK: 240 ± 767 U/L, and PCT: 1.04 ± 2.43 ng/ml. The pre-6th wave group had significantly more cases of diarrhea, while the post-6th wave group had significantly more cases of fever >37.5°C. Convulsions were observed only in the post-6th wave group, but the difference was not statistically significant. Regarding treatment, significantly more patients recovered without treatment in the pre-6th wave group (Table 4).

**Table 4:** Comparison of the pre-6th wave and post-6th wave groups for symptoms, diagnosis, and treatment.

		The pre-6th wave group *n* = 10	The post-6th wave group *n* = 111	*p*-Value
Symptom	Fever >37.5°C	3	105	<0.001
	Cough	5	37	0.289
	Convulsion	0	30	0.066
	Runny nose	3	14	0.148
	Sputum increase	1	14	>0.999
	Impaired consciousness	0	15	0.611
	Diarrhea	4	6	0.004
	Vomit	0	10	>0.999
	Sore throat	0	7	>0.999
	Headache	0	5	>0.999
	Chest pain	0	2	>0.999
	Muscle pain	0	1	>0.999
	Apnea	0	1	>0.999
	Dysgeusia	1	0	0.083
	Without symptoms	1	3	0.295
Diagnosis	Upper respiratory infection	7	64	0.521
	Complex febrile seizure	0	15	0.611
	Bronchitis	2	10	0.259
	Acute encephalopathy	0	11	0.596
	Gastroenteritis	0	6	>0.999
	Pneumonia	0	5	>0.999
	Febrile delirium	0	4	>0.999
	Simple febrile seizure	0	2	>0.999
	Myocarditis	0	1	>0.999
	Kawasaki disease	0	1	>0.999
	Myositis	0	1	>0.999
	Diabetic ketoacidosis	0	1	>0.999
Treatment	Transfusion	1	88	<0.001
	Antifebrile drug	0	60	0.001
	Expectorant drug	2	21	>0.999
	Oxygenation	1	19	>0.999
	Remdesivir	0	15	0.611
	Methylprednisolone pulse	0	11	0.596
	Intravenous immunoglobulins	0	3	>0.999
	Intubation and respirator management	0	2	>0.999
	High-flow nasal cannula	0	2	>0.999
	Dexamethasone	0	1	>0.999
	Percutaneous pacing	0	1	>0.999
	Without treatments	7	11	<0.001

### 3.4. Outcomes

In the pre-6th wave group (*n* = 10), there were 1 (10.0%) asymptomatic, 7 (70.0%) mildly-ill, 1(10.0%) moderately-ill, and 1 (10.0%) severely-ill patients. There were no critically-ill patients. The moderately-ill patient was a 2-year-old boy with an underlying 22q11.2 deletion syndrome and tetralogy of Fallot who was usually on home oxygen therapy. The patient developed acute bronchiolitis on admission; however, oxygenation was not increased until discharge. The severely-ill patient was a 3-month-old boy who had bronchiolitis with SpO2 < 94%.

In the post-6th wave group (*n* = 111), there were 3 (2.7%) asymptomatic, 69 (62.2%) mildly-ill, 14 (12.6%) moderately-ill, 22 (19.8%) severely-ill, and 3 (2.7%) critically-ill patients. The 14 patients with moderate illness had status epilepticus or seizure clusters. None of the patients presented respiratory distress. Of the 22 patients with severe illness, 13 had decreased SpO2 associated with lower respiratory tract inflammation and were subsequently administered oxygen; 8 patients had prolonged loss of consciousness and were diagnosed with acute encephalopathy; and the remaining patient had complete atrioventricular block associated with myocarditis and underwent percutaneous pacing. The three critical cases included one patient who died due to hemorrhagic shock and encephalopathy syndrome (HSES), one with hemiconvulsion-hemiplasia syndrome (HHS) and left sequelae, and one with mild encephalopathy. Patients with HSES and HHE were hospitalized during the 6th wave. The HSES patient was an 8-year-old girl with no underlying disease. The HHS patient was a 2-year-old girl with no underlying disease; she was unable to sit without support and speak at the time of discharge. The patient with mild encephalopathy was a 1-year-old boy with no underlying disease; he was hospitalized during the 8th wave. He was intubated for ventilator management due to central apnea during status epilepticus and post-ictal impaired consciousness. He was extubated the next day and discharged without sequelae.

Two patients (1.7%) had sequelae: one was the HHS patient previously described and the other was a 7-month-old boy with an underlying Apert syndrome, who was diagnosed with bronchiolitis at admission during the 8th wave and was administered with oxygen because his oxygenation had dropped to 88%. Oxygen could not be discontinued until discharge, and thus necessitating home oxygen therapy, which was considered a sequela.

There were no significant differences between the mild and severe groups in terms of sex or age (Supplementary Table S1). There were no differences in the blood test results between the mild and severe groups. However, the patient with HSES had hypercytokinaemia on admission (interleukin-6: >15,333 pg/ml, interleukin-8: >15,333 pg/ml, tumor necrosis factor-alpha: 544.3 pg/ml) had changes in platelet and coagulation system, and elevations in deviating enzymes, as follows: PLT: 18,000/µl, D-dimer: 449 µg/ml, Cr: 1.74 mg/dl, AST: 1,126 U/L, ALT: 442 U/L, LDH: 1,819 U/L. The following blood data on admission for the patient with HHS with sequelae were higher than those for other patients: CRP: 6.57 mg/dl, AST: 361 U/L, ALT: 307 U/L, LDH: 974 U/L.

Platelet counts were significantly lower in patients with neurological symptoms such as convulsions and impaired consciousness on admission than in other cases (patients with neurological symptoms vs. without neurological symptoms; median 188,000 vs. 230,000; *p*-value 0.031). No other blood test results showed significant differences.

### 3.5. Mutation strain

Mutation analysis of SARS-CoV-2 was performed on one patient in 5th wave (moderately-ill patient with 22q11.2 deletion syndrome and tetralogy of Fallot), one patient in 6th wave (patient with HSES), five patients in 7th wave (one mildly-ill patient with upper respiratory infection, three moderately-ill patients with complex febrile seizure, and one severely-ill patient with pneumoniae), four patients in 8th wave (one mildly-ill patient with upper respiratory infection, two moderately-ill patients with complex febrile seizure, and one severely-ill patient with bronchitis). SARS-CoV-2 obtained from the patient in 5th wave had *L452R* mutation. After the 6th wave, *E484A* mutation was detected in all ten SARS-CoV-2 samples.

## 4. Discussion

The number of admissions of pediatric patients with COVID-19 is increasing since the Omicron strain become endemic (10, 11). In Tochigi Prefecture, the number of pediatric patients with COVID-19 increased from the 6th wave (the Omicron epidemic period in Japan), and the number of hospitalized cases admitted to JCMCT increased significantly. In reports of admission of pediatric patients with COVID-19 before the global outbreak of Omicron strain, symptoms on admission included fever in 67%–74% cases, respiratory tract symptoms such as cough in 55%–60%, and gastrointestinal symptoms in 29%–62%, while seizure is less common at 2%–3% (12–14). In our study, in the pre-6th wave group, before the Omicron epidemic, fever, cough, and gastrointestinal symptoms manifests in 30%, 50%, and 40% of patients, respectively, while no patients had seizures. In contrast, clinical manifestations of the post-6th wave group were characterized by an increase in the number of patients with fever and febrile seizures. Similar to this study, there have been several reports of an increase in the number of pediatric COVID-19 patients with seizures during the Omicron epidemic (15–17).

In general, febrile seizures rarely occur in individuals aged >5 years (18); however, 12 patients from this age group had febrile seizures. In addition, many patients had status epilepticus or seizure clusters, corroborating the findings of a study, which reported that patients aged 6–10 years infected with the Omicron strain had status epilepticus or seizure clusters (19). Although we did not identify the mutant strain in all cases in this study, considering the epidemic situation in Japan and Tochigi Prefecture, almost all cases admitted to the JCMCT after the 6th wave were considered to have been of the Omicron strains. In fact, the causative virus of the patient with HSES in the 6th wave and some patients in the 7th and 8th wave showed the *E484A* mutation, which is characteristic of the Omicron strain. Febrile seizures, including status epilepticus and seizure clusters, should be noted in a wide range of age groups with Omicron strain infections.

Animal experiments and basic science studies suggest that the mechanisms by which COVID-19 causes neurological symptoms include direct viral invasion into the nervous system, vascular damage, and/or inflammatory reactions due to local and systemic infection (20, 21). When SARS-CoV-2 invades cells, the Omicron strain inefficiently uses the cellular protease TMPRSS2. Conversely, the Omicron strain uses cathepsin to invade the cells (22). Differences in the pathway of entry into the cells may influence varying clinical symptoms. In this study, the patient with HSES had hypercytokinemia. Examinations of cytokine markers was not performed on patient with HHS. However, the CRP, AST, ALT, and LDH levels were elevated, suggesting hypercytokinemia. As the cerebrospinal fluid examinations could not be performed in some patients due to the risk of encephalocele associated with cerebral edema, assessment of viral invasion to the central nervous system was not conducted. Examinations of cerebrospinal fluid was performed on some patients with status epilepticus; however, it remains unclear whether SARS-CoV-2 had invaded the central nerve system as PCR of SARS-CoV-2 of cerebrospinal fluid were not examined. A meta-analysis by Malik et al. suggested that decreased lymphocytes and platelets as well as increased D-dimer, CRP, PCT, CK, AST, ALT, Cr, and LDH levels were associated with severe COVID-19 (23). However, WBC, Neutr, Lymph, PLT, D-dimer, CRP, PCT, CK, AST, ALT, Cr, and LDH levels were not relevant indicators for severity assessment of pediatric patients with COVID-19 in this study. Platelet counts were significantly lower in patients with seizure and impaired consciousness. Thrombocytopenia has been reported to occur as a result of vascular damage associated with COVID-19 (24), which may have caused neurological symptoms by impairing blood flow in the brain in this study. However, it was not proven whether there was vascular damage as no imaging evaluation such as MRA, cerebral blood flow scintigraphy, or SPECT had been performed.

In Tochigi Prefecture, the number of patients aged <15 years with COVID-19 was 1,589 before the end of the 5th wave and increased approximately 32-fold to 51,081 after the 6th wave. However, the number of hospitalized patients has remained less than double. The high rate of hospitalization of pediatric patients with COVID-19 before the end of the 5th wave may be because being younger was considered a risk factor for severe illness at that time. An increasing number of reports on the clinical characteristics of patients with COVID-19 have revealed that most younger patients are asymptomatic or have mild illnesses (4, 25, 26). This may have led to a decrease in hospitalization rate after the 6th wave in Tochigi Prefecture.

In Japan, according to the Ministry of Health, Labor, and Welfare policy states, in principle, hospitalization measures for patients with COVID-19 should be taken at medical facilities in the prefecture they reside. Moderate, severe, or critical pediatric COVID-19 cases in Tochigi Prefecture are often admitted or transferred to the JCMCT. In pediatric patients with COVID-19 who had not been admitted to the JCMCT and were asymptomatic or mildly ill, there was no significant difference in the incidence of severe illness in all pediatric COVID-19 cases in Tochigi Prefecture between the pre-6th and post-6th wave groups (pre-6th wave vs. post-6th wave: 0.13% vs. 0.06%, *p* = 0.251). When restricted to the incidence of cases meeting the NIH severity criteria only, there was also no significant difference in the incidence of severe cases (pre-6th wave vs. post-6th wave: 0.13% vs. 0.02%, *p* = 0.308). As in previous reports, most pediatric COVID-19 cases were mild (4, 5, 9, 25, 26). However, the increase in the overall number of COVID-19 patients suggests that the number of patients with moderate or severe illness has also increased, resulting in the emergence of patients who died or had severe sequelae.

This study has four limitations. First, the sample size was small, as it was a single-center study, and the severity rate was assessed only in Tochigi Prefecture. The number of deaths and patients with sequelae was also small, and the statistical analysis of the background characteristics and blood tests between patients who died or had sequelae and those without sequelae could not be performed. Second, the definition of severity differs from that in the NIH criteria. This led to an increase in the number of severe cases, because the Japanese severity criteria are based on the NIH criteria. However, we were able to evaluate severe cases that did not fit the NIH criteria. Third, because virological analysis was not performed in all cases, we were unable to evaluate differences in symptoms and severity by the mutation strain. Fourth, there was no data on the bed occupancy rate of local medical facilities before and after the Omicron strain outbreak. It is possible that increased occupancy rates at local medical facilities in Tochigi Prefecture may have affected the number of admissions to the JCMCT. However, the JCMCT rarely refuses moderately, severe, or critical pediatric COVID-19 patients. The information on the severe group patients in this study is considered to be almost accurately indicative of the actual situation in Tochigi Prefecture.

## Data Availability

The original contributions presented in the study are included in the article/**Supplementary Material**, further inquiries can be directed to the corresponding author.
