# Supplementary material for: Impact of the omicron phase on a highly advanced medical facility in Japan
Source: Front Pediatr. 2023 Jul 11;11:1201825. doi: 10.3389/fped.2023.1201825 (PMC10366354; doi:10.3389/fped.2023.1201825)
Supplement: Supplementary file 1 [file Table1.pdf]

Supplemental Table 1. Comparison of the mild and severe groups regarding patient demographics and laboratory data during admission

			Mild group	Severe group	p-value
Sex		Male	40	21	0.899
		Female	40	20	
Age		Average (months old)	67	71	0.797
		±SD	70	79	
Admitted phase		pre-6th wave	8	2	0.498
		post-6th wave	72	39	
Underlying disease		With	40	16	0.252
		Without	40	25	
Laboratory data	WBC	Median (/μL)	6,700	6,000	0.647
		Range	1,900-17,300	3,100-24,200	
	Neutr	Median (/μL)	3,800	3,500	0.652
		Range	800-14,600	1,100-14,900	
	Lymph	Median (/μL)	1,200	1,300	0.622
		Range	200-7,100	200-8,700	
	PLT	Median (/μL)	226,000	204,000	0.233
		Range	33,000-893,000	18,000-445,000	
	D-dimer	Median (μg/mL)	0.8	0.5	0.346
		Range	0.5-78.9	0.5-449.2	
	CRP	Median (mg/dL)	0.47	0.50	0.579
		Range	0.01-14.42	0.03-6.57	
	Cr	Median (mg/dL)	0.32	0.30	0.154
		Range	0.14-1.05	0.15-1.74	
	AST	Median (U/L)	42	43	0.425
		Range	13-131	27-1,126	
	ALT	Median (U/L)	21	20	0.974
		Range	6-116	10-442	
	LDH	Median (U/L)	316	323	0.921
		Range	149-789	176-1819	
	CK	Median (U/L)	105	131	0.079
		Range	40-7,450	36-920	
	PCT	Median (ng/mL)	0.19	0.30	0.524
		Range	0.09-2.76	0.07-11.70	

WBC, white blood cell count; Neutr, neutrophil count; Lymph, lymphocyte count; PLT, platelet cell count; CRP, serum C-reactive protein; Cr, creatinine; AST, aspartate

aminotransferase; ALT, alanine aminotransferase; LDH, lactate dehydrogenase; CK, creatine kinase; PCT, procalcitonin
